# Identification of Four Novel Variants and Determination of Genotype–Phenotype Correlations for ABCA4 Variants Associated With Inherited Retinal Degenerations

**DOI:** 10.3389/fcell.2021.634843

**Published:** 2021-03-01

**Authors:** Qing Zhu, Xue Rui, Ya Li, Ya You, Xun-Lun Sheng, Bo Lei

**Affiliations:** ^1^Zhengzhou University People’s Hospital, Henan Provincial People’s Hospital, Zhengzhou, China; ^2^Ningxia Clinical Research Center of Blinding Eye Disease, Ningxia Eye Hospital, People’s Hospital of Ningxia Hui Autonomous Region, First Affiliated Hospital of Northwest University for Nationalities, Yinchuan, China; ^3^Henan Branch of National Clinical Research Center for Ocular Diseases, Henan Eye Institute and Henan Eye Hospital, Henan Provincial People’s Hospital, Zhengzhou, China

**Keywords:** ABCA4, inherited retinal degeneration, Stargardt diseases, cone-rod dystrophy, photoreceptor degeneration

## Abstract

**Purpose:**

The purpose of the study is to describe the genetic and clinical features of 17 patients with ABCA4-related inherited retinal degenerations (IRDs) and define the phenotype–genotype correlations.

**Methods:**

In this multicenter retrospective study, 17 patients from 16 families were enrolled, and ABCA4 gene variants were detected using targeted next-generation sequencing using a custom designed panel for IRDs. Sanger sequencing and co-segregation analysis of the suspected pathogenic variants were performed with the family members. The pathogenicities of variants were evaluated according to the American College of Medical Genetics and Genomics guidelines (ACMG). Protein structure modifications mediated by the variants were studied using bioinformatic analyses.

**Results:**

The probands were diagnosed with Stargardt disease 1 (7), cone-rod dystrophy type 3 (8), cone dystrophy (1), and retinitis pigmentosa 19 (1). Onset of symptoms occurred between 5 and 27 years of age (median age = 12.4 years). A total of 30 unique ABCA4 suspicious pathogenic variations were observed, including 18 missense mutations, seven frameshift mutations, two nonsense mutations, one canonical splice site mutation, one small in-frame deletion, and one insertion. Four novel ABCA4 variants were identified. Two novel frameshift variants, c.1290dupC (p.W431fs), and c.2967dupT (G990fs), were determined to be pathogenic. A novel missense variant c.G5761T (p.V1921L) was likely pathogenic, and another novel missense c.C170G (p.P57R) variant was of undetermined significance. All ABCA4 variants tested in this study inordinately changed the physico-chemical parameters and structure of protein based on *in silico* analysis.

**Conclusion:**

ABCA4-related IRD is genetically and clinically highly heterogeneous. Four novel ABCA4 variants were identified. This study will expand the spectrum of disease-causing variants in ABCA4, which will further facilitate genetic counseling.

## Introduction

Inherited retinal degenerations (IRDs) are a group of blinding diseases that cause severe impairments of visual functions such as visual acuity and visual field. More than 270 disease-causing genes are associated with IRD (RetNet)^1^. Among them, ABCA4 is the most frequently identified gene (Kim et al., 2019; Pontikos et al., 2020). Mutations in the ABCA4 gene can cause various retinal diseases such as autosomal recessive (ar)-Stargardt disease (STGD1, OMIM # 248200), ar-cone-rod dystrophy (CORD3, OMIM # 604116), ar-cone dystrophy (COD), ar-retinitis pigmentosa (RP19, OMIM # 601718), and age-related macular degeneration-2 (ARMD2, OMIM # 153800) (van Driel et al., 1998; Baum et al., 2003; Burke and Tsang, 2011; Xin et al., 2015; Wang et al., 2018; Koyanagi et al., 2019).

ATP-binding cassette subfamily A member 4, denoted as ABCA4 (OMIM #601691), was first cloned by Allikmets et al. (Allikmets et al., 1997). The gene contains 50 exons, and it maps to position 22.1 (1p22.1) of the short (p) arm of chromosome 1 (Nasonkin et al., 1998). ABCA4 gene encodes a transmembrane protein that is exclusively expressed in photoreceptors and retina pigment epithelial cells (Lenis et al., 2018). The protein is composed of two non-equivalent tandem halves, and each half contains six transmembrane helices, a glycosylated extracytoplasmic domain (ECD) in the endolysosomes or disks, and a nucleotide binding domain in the cytoplasm. ATP-binding cassette transporter unidirectionally flips various compounds obtained from enzyme-catalyzed reactions of the visual cycle to the cytoplasm using the energy released from ATP hydrolysis (Kos and Ford, 2009; Quazi et al., 2012; Lenis et al., 2018). The misfolding and loss of functional activity of the transporter, caused by gene mutations, lead to accumulation of toxic substances such as all-trans-retinal and 11-cis-retinal in the outer segment of photoreceptor cells. Following diurnal phagocytosis of the distal outer segment of photoreceptors, excessive deposition of secondary toxic products, such as bisretinoid, eventually leads to the death of retinal pigment epithelium (Young and Bok, 1969; Weng et al., 1999; Quazi and Molday, 2014; Lenis et al., 2018). Different ABCA4 mutations lead to a broad range of IRD phenotypes (Rozet et al., 1998; van Driel et al., 1998; Xu et al., 2014; Garces et al., 2018). Phenotype severity mainly depends on the degree of influence of variations on protein functions (Fujinami et al., 2015).

Rapid advances in high-throughput next-generation sequencing technology have enabled an efficient and credible detection of gene mutations (Glöckle et al., 2014; Rong et al., 2018). Currently, 1,467 ABCA4 gene variants, containing a broad spectrum of IRD phenotypes, are present in the Human Gene Mutation Database^2^ (updated on April, 2019). However, the pathogenicities of numerous reported variants have not been elucidated yet, making their accurate clinical diagnoses difficult, let alone those of novel variants. Additionally, the analyses of genotype–phenotype correlations for the highly heterogeneous variants and clinical features are a challenge.

Therefore, we conducted this retrospective study to determine the pathogenicities of ABCA4 gene variants and novel genotype–phenotype correlations.

## Materials and Methods

### Ethical Approval

This multicenter retrospective study was conducted at the Henan Eye Hospital and the Ningxia Eye Hospital. The study was approved by the Medical Ethics Committees of both institutions, and it was conducted in accordance with the 1975 Declaration of Helsinki guidelines. Written informed consent was obtained from all included subjects or their guardians.

### Subjects

Seventeen patients with retinal degeneration, carrying the ABCA4 gene variants and belonging to 16 unrelated Chinese families, were recruited in the study from ophthalmic clinics. Of these, 11 patients were enrolled from Henan Eye Hospital (B1–11) and six patients from Ningxia Eye Hospital (A1–6). Family history, if available, was obtained from the patients and unaffected family members. Detailed clinical data, including age of onset, disease duration, best-corrected visual acuity (BCVA), and result of color vision testing, fundus photography, fundus autofluorescence imaging, fundus fluorescein angiography, optical coherence tomography, and full-field electroretinogram, were collected. Diagnostic criteria were adapted from previous studies (Fishman, 1976; Hamel, 2007; Sahel et al., 2014; Aboshiha et al., 2016; Verbakel et al., 2018). Retina specialists performed phenotype subgroup classification on all 17 included subjects.

### Mutation Screening

Whole genomic DNA was extracted from the peripheral blood of the subjects using the TIANamp Blood DNA kit DP318 (TIANGEN, Beijing, China) according to the manufacturer’s protocol. A custom-designed posterior segment gene detection kit (PS400), containing 376 known causative IRD genes, their coding exons, flanking intronic sequences (50 bp), and all known intron mutations, was used for mutation screening. Sequence reads were aligned to the reference human genome (GRCh37/hg19) from the UCSC Genome Browser (Kent et al., 2002)^3^ using the XYGeneRanger2.0 software. Suspicious disease-relevant gene variants were routinely confirmed using Sanger sequencing, and co-segregation analyses were conducted for all affected families.

### *In silico* Analysis

All variants were classified according to the American College of Medical Genetics and Genomics (ACMG) standards and guidelines (Richards et al., 2015). To exclude the possibility of non-pathogenic polymorphism, the frequency of variations in the healthy control population was determined using the 1,000 Genomes Project (1,000 Genomes)^4^ and the Genome Aggregation Database^5^. A variant was classified as benign if its minor allele frequency was ≥ 0.005. Prediction algorithms of the programs Polyphen-2^6^, SIFT^7^, PROVEAN^8^, CADD^9^, FATHMM (Shihab et al., 2013)^10^, and MutationTaster^11^ were used to test variants for disease relevance. Clustal Omega^12^ was used to analyze the conservative loci. For the variants of uncertain significance. Human Splicing Finder (Desmet et al., 2009)^13^ and Project HOPE (Venselaar et al., 2010)^14^ were used to predict the splicing defects and structural effects of variants.

## Results

Clinical characteristics of patients are summarized in Table 1. Seven subjects were diagnosed with STGD1 (7/17, 41.2%), eight with CORD3 (8/17, 47%), one with RP19 (1/17, 5.9%), and one with COD (1/17, 5.9%). Hypopsia was the chief complaint in all 17 patients. The age of onset of diseases in patients ranged from 5 to 27 years with a median age of 12.4 years. The BCVA of the most recent evaluation ranged from 0.3 to 0.02 across the group. One RP19 patient (A1) and three patients with cone/cone–rod dystrophy had color vision disorders (A2, A4, and A5), all others possessed normal color vision.

**TABLE 1:** Clinical characteristics, including ABCA4 variations, of the 17 patients in the study.

Patient	Diagnosis	Onset (y)	DD (y)	BCVA	ffERG^b^		ABCA4 Variation(s)
		
				OD/OS	Rods	Cones	
A1	RP19	27	7	0.3/0.3 +	NA	NA	c.G2473A; c.G673A
B1	CORD3	13	25	0.05/0.05	↓↓↓	↓↓↓	c.A2894G; c.2063dupA
B2	CORD3	20	13	0.04/0.05	↓↓	↓↓↓	c.A2894G; c.1290dupC
B3	CORD3	5	2	0.05/0.05	↓↓↓↓	↓↓↓↓	c.2967dupT; c.T4748C
B4	CORD3	17	10	0.06/0.05	↓	↓↓	c.C3322T; c.G1648A
B5	CORD3	12	6	0.05/0.02	↓	↓↓	c.A2894G; c.G3106A; c.A983T
A2	CORD3	9	1	0.15/0.15	↓↓	↓↓	1761–2A > G; c.C5512T
A3	CORD3	5	17	0.06/0.02	↓↓↓↓	↓↓↓↓	c.170_171insAA; c.C170G
A4	CORD3	10	10	0.05/0.15	NA	NA	c.1006delT; c.618_619insAAGGACATCGCCTGCAGC
A5	COD	13	4	0.15/0.12	↓	↓	c.A2894G; c.A1034G
B6^a^	STGD1	12	5	0.05/0.05	↓↓	↓↓↓	c.T1035G; c.A1034C
B7^a^	STGD1	12	0	0.05/0.05	↓	↓↓	c.T1035G; c.A1034C
B8	STGD1	11	1	0.05/0.07	NA	NA	c.C6118T; c.101_106del
B9	STGD1	6	10	0.04/0.05	NA	NA	c.4203delC; c.C203T
B10	STGD1	10	2	0.08/0.1	Normal	↓↓↓↓	c.1561delG; c.1760G > A
B11	STGD1	12	10	0.08/0.06	NA	NA	c.C5593T; c.G5761T; c.G3106A; c.C5318T
A6	STGD1	17	4	0.12/0.12	Normal	↓	c.C4070T; c.C4070T

*y, years; DD, disease duration; OD, right eye; OS, left eye; ffERG, full-field electroretinogram; RP19, retinitis pigmentosa19; CORD3, cone–rod dystrophy 3; COD, cone dystrophy; STGD1, Stargardt disease 1; NA, not available; ↓↓↓↓, severely attenuated; ↓↓↓, moderate-severely attenuated; ↓↓, moderately/mild-moderately attenuated; ↓,mildly attenuated.*

*^*a*^Patients B4 and B5 are siblings.*

*^*b*^The right eye.*

**FIGURE 1:**
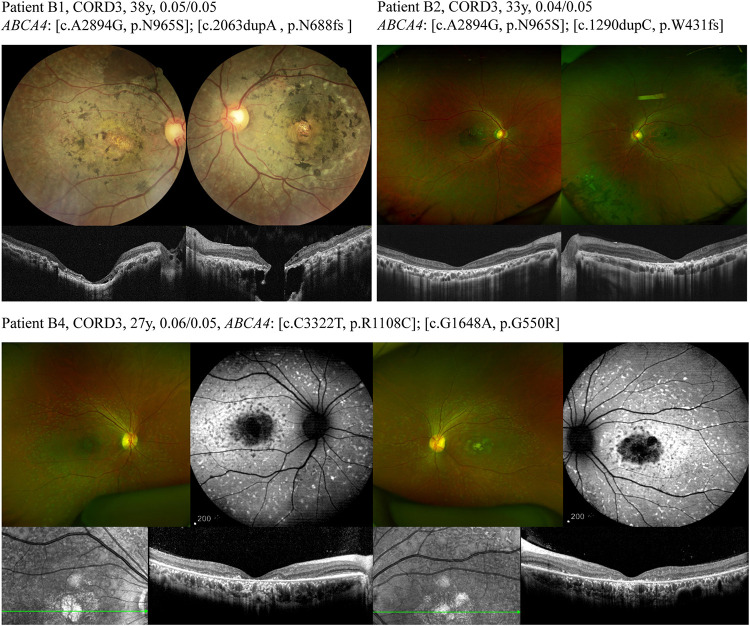
Different ABCA4 mutations lead to a broad range of phenotypes of cone-rod dystrophy. Fundus of patient B1 exhibited a relatively pale and blonde appearance due to exposure of the underlying sclera and nummular pigmentary deposition in the fovea of both eyes. The fundus retinal pigmentation in patient B2 was mainly distributed in the mid-periphery. Wide-field color fundus photograph of patient B4 demonstrated diffuse pisciform flecks throughout the posterior pole, macular atrophy, and no obvious retinal pigmentation.

**FIGURE 2:**
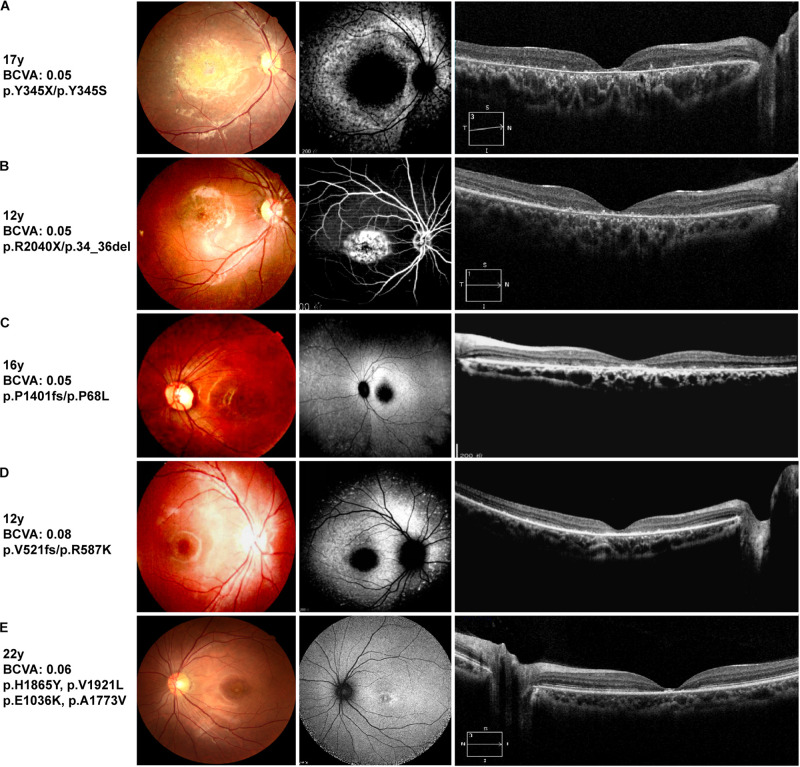
Representative clinical features of STGD1 patients. Left: color fundus images, center: retinal autofluorescence imaging or fundus photography, right: optical coherence tomography images. **(A)** Patient B6, **(C)** Patient B9, and **(D)** Patient B10 harboring a compound heterozygous truncating mutation and missense mutation. Fundus autofluorescence shows hypo-autofluorescence at the fovea with different sizes of macula atrophy. Optical coherence tomography shows atrophy around the fovea of STGD1 with the most severe degeneration for patient B6. **(B)** Center-fluoresce in angiography of the right eye (AV-transit phase) of patient B8 showing a window defect in the area of bull’s eye atrophy. The retinal deposits exhibit hyperfluorescence. **(E)** Fundus autofluorescence of patient B11, an individual with four ABCA4 missense mutations, showing a parafoveal hyperfluorescent ring.

Genotypic and phenotypic variations were observed in three patients, and others possessed a typical ABCA4-associated disease phenotype. Patient A1 was asymptomatic until 27 years of age. The patient was diagnosed with retinitis pigmentosa along with BCVA of 0.3 bilaterally. The patient carried heterozygous mutations for both ABCA4 (NM_000350: c.G2473A, p.G825R and c.G673A, p.V225M) and AHI1 (NM_001134830: c.G3267A, p.W1089X and c.T1979G, p.L660R), and color-vision testing revealed errors in the red–green axis. The cone–rod dystrophy patient (B1), who carried compound heterozygous variants (NM_000350: c.A2894G, p.N965S and c.2063dupA, p.N688fs) in the ABCA4 gene, complained of visual defects with strabismus for 25 years. Her latest BCVA was 0.05 bilaterally. Retinal degenerative features were observed in the fundus, and exposure of the underlying sclera was observed in the fovea of both eyes. Spectral domain optical coherence tomography scans through the fovea revealed retinal rupture with a subretinal cavitation in the fovea of the left eye (Figure 1). Patient B4, with two heterozygous missense mutations in ABCA4, had blurred vision in both eyes for 10 years, and fundus autofluorescence exhibited diffuse patches of macular atrophy with nascent fleck development in the mid-periphery (Figure 1), which is similar to the ABCA4 phenotype in the early stage of rapid-onset chorioretinopathy (Tanaka et al., 2018). Additionally, the STGD1 patients demonstrated typical fundus manifestations of macular atrophy with miscellaneous sizes (Figure 2).

**FIGURE 3:**
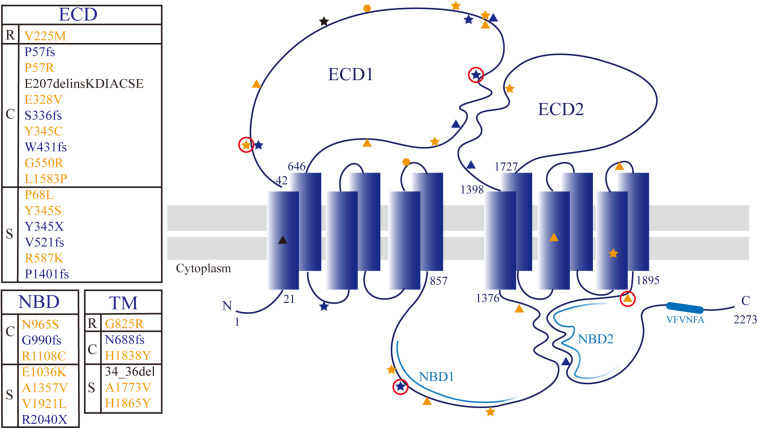
Topological model of ABCA4 protein showing the distribution of detected mutations. The textbox on the left of the model shows correspondence between the locus and phenotype (R: RP19; C: CORD3/COD; S: STGD1). The sites associated with RP19, CORD3/COD, and STGD1 phenotypes are denoted with hexagon, pentagram, and triangle, respectively. Missense mutation, protein truncating mutation, and non-frame-shift deletion are colored yellow, blue, and black, respectively. The novel variants are circled in red.

We detected a total of 30 unique ABCA4 variants, including missense mutations (18), frameshift mutations (7), non-sense mutations (2), a splice site mutation (1), a small deletion (1), and an insertion (1). The 17 individuals, including one sibling pair, with at least two variants in the ABCA4 gene belonged to 16 families. Of the 30 rare ABCA4 variants, 24 were classified as pathogenic or likely pathogenic, and six were of uncertain significance. The pathogenicity of other mutated genes, except for the AHI1 in patient A1, was ruled out by phenotypic analysis. Frameshift mutations [c.1290dupC (p.W431fs) and c.2967dupT (G990fs)] and missense mutations [c.C170G (p.P57R) and c.G5761T (p.V1921L)] were novel unpublished variants (Table 2). The missense mutation c.A2894G (p.N965S) was detected four times without any exception in patients with cone/cone–rod dystrophy. The amino acid changes were primarily concentrated in ECD1 (47%, 14/30), and the amino acid variants were randomly distributed (Figure 3). Moreover, the HOPE online software revealed that all missense variants in this study inordinately changed the physico-chemical parameters or structure of ABCA4 (Appendix 1).

**TABLE 2:** Pathogenicity analyses of ABCA4 (NM_000350) variants in the 17 Chinese patients.

**Online Prediction**													

**Proband**	**Exon/Intron**	**Nucleotide change**	**Protein Variant**	**Polyphen^c^ (Score)**	**SIFT (Score)**	**PROVEAN (Score)**	**CADD_Phred (Score)^d^**	**FATHMM (Score)**	**Mutation Taster**	**MAF in gnomAD^e^(%)**	**MAF in 1000G**	**Source^f^**	**Category**
A1	16	c.G2473A	p.G825R	B (0.022)	T(0.411)	Neutral (−1.139)	12.55	Damaging (−2.4)	DC	0.0208	NA	rs368367104	VUS
	6	c.G673A	p.V225M	PrD (0.965)	D(0.006)	Neutral (−1.336)	17.34	Damaging (−2.9)	DC	0.2807	−	Koyanagi et al., 2019	VUS
B1	19	c.A2894G	p.N965S	PrD (0.961)	D(0.012)	Deleterious (−4.624)	25.9	Damaging (−3.59)	DC	0.04009	NA	Rosenberg et al., 2007	P
	14	c.2063dupA	p.N688fs	−	−	−	−		DC(NMD)	NA	NA	Xu et al., 2014	P
B2	19	c.A2894G	p.N965S	PrD (0.961)	D(0.012)	Deleterious (−4.624)	25.9	Damaging (−3.59)	DC	0.04009	NA	Rosenberg et al., 2007	P
	10	c.1290dupC	p.W431fs	−	−	−	−		DC(NMD)	NA	NA	Novel	P
B3	20	c.2967dupT	p.G990fs	−	−	−	−		DC(NMD)	NA	NA	Novel	P
	33	c.T4748C	p.L1583P	PoD (0.878)	D(0.002)	Deleterious (−4.466)	17.4	Damaging (−2.89)	DC	0.01087	NA	Kim et al., 2019	VUS
B4	22	c.C3322T	p.R1108C	PrD (0.937)	D(0.000)	Deleterious (−7.002)	25.8	Damaging (−3.73)	DC	0.005013	0.0599	Rozet et al., 1998	LP
	12	c.G1648A	p.G550R	PoD (0.523)	D(0.003)	Deleterious (−5.955)	18.07	Damaging (−5.12)	DC	0.000	NA	Shroyer et al., 2001	LP
B5	19	c.A2894G	p.N965S	PrD (0.961)	D(0.012)	Deleterious (−4.624)	25.9	Damaging (−3.59)	DC	0.04009	NA	Rosenberg et al., 2007	P
	21	c.G3106A	p.E1036K	B (0.010)	T(0.681)	Neutral (−0.820)	7.787	Damaging (−3.16)	DC	0.01631	NA	Nasonkin et al., 1998	LP
	8	c.A983T	p.E328V	B (0.236)	D(0.014)	Deleterious (−4.199)	15.46	Damaging (−2.83)	DC	0.005437	NA	Rivera et al., 2000	VUS
A2	IVS12	1761−2A > G	−	−	−	−	21.9	−	Acceptor splice sites abolished	0.005472	NA	Jiang et al., 2016	P
	39	c.C5512T	p.H1838Y	PrD (0.989)	D(0.001)	Deleterious (−4.625)	27.3	Damaging (−2.2)	DC	NA	NA	Lewis et al., 1999	LP
A3	3	c.170_171insAA	p.P57fs	−	−	−	−		DC(NMD)	NA	NA	Rong et al., 2018	P
	3	c.C170G	p.P57R	PoD(0.602)	D(0.006)	Deleterious (−5.667)	23	Damaging (−5.02)	DC	NA	NA	Novel	VUS
A4	8	c.1006delT	p.S336fs	−	−	−	−	−	DC(NMD)	NA	NA	Hu et al., 2019	P
	6	c.618_619ins AAGGACAT CGCCTGCAGC	p.E207delins KDIACSE	−	−	Deleterious (−7.314)	−	−	p	NA	NA	Tian et al., 2016	LP
A5	19	c.A2894G	p.N965S	PrD (0.961)	D(0.012)	Deleterious (−4.624)	25.9	Damaging (−3.59)	DC	0.04009	NA	Rosenberg et al., 2007	P
	8	c.A1034G	p.Y345C	PoD(0.808)	D(0.002)	Deleterious (−4.541)	21.6	Damaging (−2.77)	DC	NA	NA	rs1417184535	LP
B6a	8	c.T1035G	p.Y345X	−	−	−	36	−	DC(NMD)	NA	NA	Wang et al., 2018	P
	8	c.A1034C	p.Y345S	B (0.035)	D(0.003)	Deleterious (−4.529)	16.5	Damaging (−2.7)	DC	NA	NA	Jiang et al., 2016	LP
B7a	8	c.T1035G	p.Y345X	−	−	−	36	−	DC(NMD)	NA	NA	Wang et al., 2018	P
	8	c.A1034C	p.Y345S	B (0.035)	D(0.003)	Deleterious (−4.529)	16.5	Damaging (−2.7)	DC	NA	NA	Jiang et al., 2016	LP
B8	44	c.C6118T	p.R2040X	−	−	−	36	−	DC(NMD)	0.01504	NA	Baum et al., 2003	P
	2	c.101_106del	p.34_36del	−	−	Deleterious (−16.717)	−	−	DC	0.02719	NA	Huang et al., 2018	P
B9	28	c.4203delC	p.P1401fs	−	−	−	−	−	DC(NMD)	NA	NA	Jiang et al., 2016	P
	3	c.C203T	p.P68L	PoD(0.826)	D(0.003)	Deleterious (−6.677)	31	Damaging (−7.45)	DC	0.000	NA	Rivera et al., 2000	LP
B10	12	c.1561delG	p.V521fs	−	−	−	−	−	DC(NMD)	0.005437	NA	Xin et al., 2015	P
	12	c.1760G > A	p.R587K	B (0.270)	T(0.510)	Neutral (−1.224)	10.66	Damaging (−4.03)	DC	NA	NA	Fujinami et al., 2015	LP
B11	40	c.C5593T	p.H1865Y	B (0.006)	T(0.903)	Neutral (−0.888)	0.006	Damaging (−2.63)	p	0.1631	−	rs201707267	VUS
	41	c.G5761T	p.V1921L	PoD(0.625)	D(0.003)	Deleterious (−2.696)	27.9	Damaging (−3.66)	DC	NA	NA	Novel	LP
	21	c.G3106A	p.E1036K	B (0.01)	T(0.681)	Neutral (−0.820)	7.787	Damaging (−3.16)	DC	0.01631	NA	Nasonkin et al., 1998	LP
	38	c.C5318T	p.A1773V	PoD(0.892)	D(0.044)	Deleterious (−3.030)	21.3	Damaging (−1.73)	DC	0.005437	NA	Chacon-Camacho et al., 2013	P
A6	27	c.C4070T	p.A1357V	PoD(0.889)	D(0.001)	Deleterious (−3.710)	29.4	Damaging (−3.36)	DC	NA	NA	Noupuu et al., 2016	LP
	27	c.C4070T	p.A1357V	PoD(0.889)	D(0.001)	Deleterious (−3.711)	29.4	Damaging (−3.36)	DC	NA	NA	Noupuu et al., 2016	LP

**MAF, minor allele frequency; B, benign; PrD, probably damaging; PoD, possibly damaging; T, tolerated; D, deleterious (<0.05); DC, disease causing; NMD, nonsense-mediated decay; p, polymorphism; NA, not appliable; −, not done; VUS, uncertain significance; LP, likely pathogenic; P, pathogenic. aSiblings. cHumVar. dThe threshold level was set to 10. eRefer to the frequency in East Asian population. fPublication or in dbSNP.**

## Discussion

**FIGURE 4:**
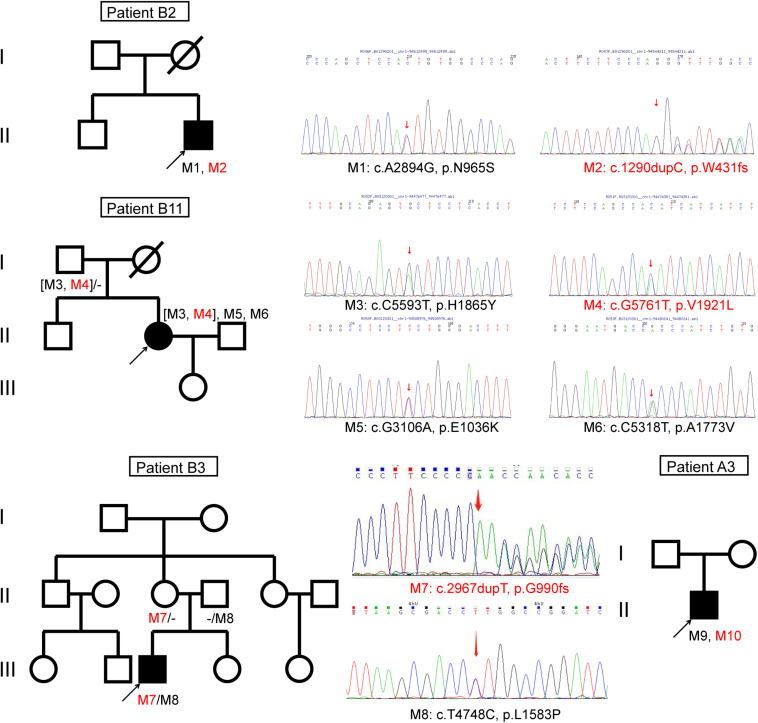
Pedigrees of four Chinese families with inherited retinal disorders harboring the ABCA4 novel variants (red font).

We identified four novel variants of the ABCA4 gene (Figure 4). According to ACMG standards and guidelines, two novel ABCA4 frameshift variants were pathogenic, one missense variant c.G5761T (p.V1921L) was likely pathogenic, and another missense variant c.C170G (p.P57R) was a variant of uncertain significance (**Appendix 2**).

The well-conserved VFVNFA motif (amino acids 2244–2249) present within the C-terminal of ABCA4 enables folding of the polypeptide into a functionally active protein (Zhong et al., 2009). Deletion of the C-terminal domain, including the VFVNFA motif, leads to a loss of ABCA4 functions, such as N-retinylidene-phosphatidylethanolamine substrate binding, ATP photoaffinity labeling, retinal-stimulated ATPase activity, and nucleotide binding domain interactions (Zhong et al., 2009; Patel et al., 2019). All frameshift and nonsense mutations of ABCA4 caused a removal of the C-terminal conserved sequence VFVNFA in the polypeptide. The downstream sequence of the VFVNFA motif also plays a role in regulating the functions of ABCA4 (Zhong et al., 2009). Protein-truncating mutations in ABCA4 leads to truncated protein products or protein loss due to nonsense-mediated RNA decay (Khajavi et al., 2006; Makelainen et al., 2019). Hence, the frameshift and nonsense ABCA4 mutations, which are defined as pathogenic according to ACMG guidelines, may have a great impact on gene expression and protein functions (Table 2). The allele frequencies of the small deletion mutation c.101_106del (p.34_36del) in two Chinese patient cohorts were 3.1% (Jiang et al., 2016) and 10.5% (Hu et al., 2019). This mutation, located in the transmembrane domain, is predicted to break the transmembrane helical structure, leading to ABCA4 transport dysfunction. The variant c.618_619insAAGGACATCGCCTGCAGC (p.E207delinsKDIACSE) inserted six amino acids in ECD1 of ABCA4, which was predicted to make ECD1 more hydrophobic and alter the protein structure. The mutation is associated with STGD1 disease (Tian et al., 2016).

The majority of identified ABCA4 sequence variants are missense mutations. Therefore, analysis of ABCA4-associated retinal degeneration is difficult. Additionally, determination of the pathogenicity of a particular variant is difficult. Testing the phenotypic associations of all amino acid substitutions along with experimental characterizations of their effects on protein functions would be extremely expensive and time consuming. Therefore, an online study of their putative effects would be conducive to prioritize the most probable disease-causing variations associated with these diseases. To further study the pathogenesis and possibility of amino acid substitutions that damage the protein, we analyzed the effects of variants on the protein using an online software (**Appendix 1**). Biochemical studies on ABCA4 missense mutations have reported that insertion of charged amino acids in transmembrane domains leads to a decrease in protein expression (Sun et al., 2000). Apart from the variations located in nucleotide-binding domains (Sun et al., 2000; Ahn et al., 2003), the missense mutations of ABCA4 gene, in or close to the transmembrane region, causing protein conformational changes can impair azido-ATP labeling to the nucleotide binding domains (Sun et al., 2000; Garces et al., 2018). Three cone–rod dystrophy patients (B1, B2, and B5) and one cone dystrophy patient (A5) harbored the missense mutation c.A2894G (p.N965S). The prevalence of the c.A2894G (p.N965S) mutation was higher in STGD1-affected individuals than in controls (Rosenberg et al., 2007; Jiang et al., 2016; Hu et al., 2019). Therefore, we hypothesized that patients carrying c.A2894G (p.N965S) might more likely manifest cone–rod dystrophy as the disease progresses, although the early stage may be an STGD1-like phenotype. The mutation, which is a replacement of asparagine with serine, resides in the conserved WalkerA sequence of nucleotide binding domain 1, which participates in nucleotide binding (Tsybovsky et al., 2013). An *in vivo* experiment concluded that p.N965S mutation causes a loss of substrate-dependent ATPase activity of ABCA4 and protein misfolding (Molday et al., 2018).

Patient B11 harbored four unique ABCA4 gene missense variants. The variant c.C5593T (p.H1865Y) and a novel variant c.G5761T (p.V1921L) were inherited from his healthy father. The missense variant c.G5761T (p.V1921L), adjacent to the transmembrane region, was likely pathogenic according to ACMG standards (Table 2). The variant c.C5593T (p.H1865Y), which has not been reported to date, was predicted as “tolerated” using a five-function prediction software. The other two missense mutations c.G3106A (p.E1036K) and c.C5318T (p.A1773V) were classified as STGD1 disease-related variants (Rivera et al., 2000; Chacon-Camacho et al., 2013). The variant c.C5318T (p.A1773V) was conserved and located in the transmembrane domain of the ABCA4 protein. The allele frequency of this mutation in an STGD1 patient cohort is significantly higher than that in the control group (Chacon-Camacho et al., 2013). According to bioinformatic analysis, it was disease causing. Homozygous p.Ala1773Val mutation leads to a severe phenotype, which is similar to that of patients harboring null ABCA4 variants (Lopez-Rubio et al., 2018). However, the fundus autofluorescence of patient B11 did not reveal extensive retinal atrophy (Figure 2), which may be related to the heterozygous state of the c.C5318T (p.A1773V) mutation. The trans variant may be a “milder” mutation. Patient A6 harbored a highly conserved ABCA4 homozygous mutation c.C4070T (p.A1357V), which was revealed to be consistently pathogenic using the six-function software. A patient harboring compound heterozygous variants of p.A1357V and p.G1961E has been reported to possess a foveal sparing (Noupuu et al., 2016). The p.G1961E mutation is usually associated with a late onset and mild phenotype (Lewis et al., 1999; Burke et al., 2012). Patient A6 was asymptomatic until 17 years of age, and the phenotype was milder compared with that of other patients. Considering this, we hypothesized that p.A1357V is not a severe mutation.

Genetic studies revealed that the patient A1 harbored variations in both ABCA4 and AHI1 genes. The two missense mutations c.G2473A (p.G825R) and c.G673A (p.V225M), located in ABCA4, were two variants of uncertain significance according to ACMG standards. Based on the analyses of the ABCA4 protein structure, p.G825R was located in the torsion angles of the transmembrane domain, which will force the local backbone into an incorrect conformation to disturb the local structure. p.V225M was located in ECD1, which will slightly destabilize the local conformation. The c.G3267A (p.W1089X) and c.T1979G (p.L660R) variants were identified in the AHI1 gene. The nonsense mutation was located in the SH3 domain of AHI1, and the missense mutation was located in the WD40 domain. Missense mutations in the WD40 domain of the AHI1 gene can underlie non-syndromic retinitis pigmentosa (Nguyen et al., 2017). ABCA4-related retinitis pigmentosa in patients is frequently caused by combinations of ABCA4 null mutations such as frameshift and splicing site mutations (van Driel et al., 1998; Shroyer et al., 2001; Huang et al., 2018). Thus, we suspect that the phenotype of patient A1 with retinitis pigmentosa may be a result of variations in both ABCA4 and AHI1 genes, which highlights the genotypic variability associated with ABCA4-related retinitis pigmentosa.

The missense mutations c.C203T (p.P68L), c.A1034G (p.Y345C), c.A1034C (p.Y345S), c.C5512T (p.H1838Y), c.G1648A (p.G550R), c.C4070T(p.A1357V), and c.C3322T (p.R1108C) have been conserved during evolution. The minor allele frequencies of these mutations in 1,000 Genomes and gnomAD database were less than 0.005. They were predicted as “deleterious” using SIFT, PROVEAN, CADD, FATHMM, and MutationTaster. Considering the lack of functional experiments to verify the effect of mutant residues and based on the results of HOPE, we classified them as “likely pathogenic” in accordance with the ACMG guidelines (Table 2). The missense mutations c.A983T (p.E328V) and c.T4748C (p.L1583P), and novel mutation c.C170G (p.P57R) were all located in ECD1 of ABCA4. The biological functions of the ECDs of ABCA4 were unknown, and the results of computer prediction were contradictory. The clinical significance of the three missense mutations was unclear.

According to the genotype–phenotype correlation model proposed by van Driel et al., the severity of the phenotype of ABCA4-related diseases is inversely proportional to the residual function of the mutant protein (van Driel et al., 1998). Hence, we could evaluate the severity and prognosis of ABCA4-related diseases using the genotype. Because the protein-truncating mutations cause nearly a complete loss of protein function, the residual function of protein caused by trans mutations primarily determines the severity of the phenotype. In this study, all 11 patients (B1, B2, B3, A3, A4, B6, B7, B8, B9, and B10) carried a severe ABCA4 gene variant, including frameshift mutations, nonsense mutations, and the missense mutation c. C5318T (p. A1773V). Additionally, the severity of the phenotype was related to the course of the disease; therefore, follow-up was highly recommended.

ABCA4-related retinal degeneration is genetically and clinically heterogeneous. The high allelic heterogeneity makes molecular genetic analysis of ABCA4-associated retinal disease challenging. We described the findings of mutational profiling of the ABCA4 gene and related clinical phenotypes, and we predicted the pathogenicities of newly discovered variants, which will expand the spectrum of disease-causing variants in ABCA4, and further facilitate genetic counseling. For the variants of unknown significance due to limited data, further experimental verification is needed to provide new insights into the molecular mechanisms of the disease, which may help in the development of precision medicine.

## Data Availability Statement

The datasets presented in this study can be found in online repositories. The names of the repository/repositories and accession number (s) can be found below: https://databases.lovd.nl/shared/individuals?search_owned_by_=%3D%22Qing%20Zhu%22.

## Ethics Statement

The studies involving human participants were reviewed and approved by The Medical Ethics Committee of People’s Hospital of Ningxia Hui Autonomous Region and Henan Provincial Eye Hospital. Written informed consent to participate in this study was provided by the participants’ legal guardian/next of kin. Written informed consent was obtained from the individual (s), and minor (s)’ legal guardian/next of kin, for the publication of any potentially identifiable images or data included in this article.

## Author Contributions

BL and X-LS conceived and designed this study, directed the work, and finalized the manuscript. QZ and XR collected the clinical samples and clinical data. YL, YY, and QZ analyzed the sequencing data. QZ collected the information and drafted and revised the manuscript. All authors contributed to the article and approved the submitted version.

## Conflict of Interest

The authors declare that the research was conducted in the absence of any commercial or financial relationships that could be construed as a potential conflict of interest.

## Footnotes

1 https://sph.uth.edu/retnet/

2 http://www.hgmd.cf.ac.uk/ac/index.php

3 http://genome.ucsc.edu/

4 http://browser.1000genomes.org

5 http://gnomad.broadinstitute.org/

6 http://genetics.bwh.harvard.edu/pph2

7 http://sift.jcvi.org

8 http://provean.jcvi.org/index.php

9 https://cadd.gs.washington.edu/

10 http://fathmm.biocompute.org.uk/

11 http://www.mutationtaster.org

12 https://www.ebi.ac.uk/Tools/msa/clustalo/

13 http://www.umd.be/HSF/

14 https://www3.cmbi.umcn.nl/hope

## Abbreviations

IRDs: inherited retinal degenerations
ar: autosomal recessive
ACMG: American College of Medical Genetics and Genomics
ECD: extracytoplasmic domain
BCVA: best-corrected visual acuity.
