# Supplementary material for: Identification of Four Novel Variants and Determination of Genotype–Phenotype Correlations for ABCA4 Variants Associated With Inherited Retinal Degenerations
Source: Front Cell Dev Biol. 2021 Mar 1;9:634843. doi: 10.3389/fcell.2021.634843 (PMC7957020; doi:10.3389/fcell.2021.634843)
Supplement: Supplementary file 1 [file Data_Sheet_1.docx]

Supplementary Material

# Supplementary Tables

| **Appendix 1:** The Effect of missense mutations on protein structure and physico-chemical parameters changes of mutant protein compared to wildtype in this study. | | | | | | |
| --- | --- | --- | --- | --- | --- | --- |
| **Type** | **Domain** | **Molecular weight** | **Theoretical pI** | **Grand average of hydropathicity** | **Conservation** | **Effects of variation on protein structure** |
| Wildtype | - | 255943.83 | 5.89 | -0.055 | YES | - |
| P57R | ECD1 | 256002.90 | 5.91 | -0.056 | YES | Disturbs the special backbone conformation caused by very rigid Prolines |
| P68L | ECD1 | 255959.87 | 5.89 | -0.052 | YES | Disturbs the special backbone conformation caused by very rigid Prolines |
| V225M | ECD1 | 255975.89 | 5.89 | -0.056 | YES | Loss of the cysteine bridge, which is important for stability of the protein. And prefers to change the predicted β-strand of the wild-type |
| E328V | ECD1 | 255913.85 | 5.91 | -0.051 | YES | Loss of hydrogen bonds and/or disturb correct folding |
| Y345S | ECD1 | 255867.73 | 5.89 | -0.054 | YES | Prefers to be in another secondary structure that is different from the predicted β-strand of the wild-type |
| Y345C | ECD1 | 255883.79 | 5.89 | -0.053 | YES | Prefers to be in another secondary structure that is different from the predicted β-strand of the wild-type |
| G550R | ECD1 | 256042.97 | 5.91 | -0.056 | YES | Abolishes the most flexible wild-type residue, which might be necessary for the protein's function |
| R587K | ECD1 | 255915.82 | 5.89 | -0.054 | YES | The base substitution may corrupt functional acceptor splice site, which may give rise to protein products truncated or NMD |
| L1583P | ECD2 | 255927.79 | 5.89 | -0.057 | YES | Disrupts the α-helix of the wild-type residue which can have severe effects on the structure of the protein |
| N965S | NBD1 | 255916.80 | 5.89 | -0.053 | YES | Loss of interactions with the nucleotide. The new residue is not in the correct position to make the same hydrogen bond as the original wild-type residue did |
| E1036K | NBD1 | 255942.89 | 5.93 | -0.055 | YES | Might affect the ability to contact with other molecules or domains |
| R1108C | NBD1 | 255890.78 | 5.86 | -0.051 | YES | Disturb the domain and abolish its function, loss of the salt bridge with Aspartic Acid at position 1048 made by the wild-type residue |
| A1357V | - | 255971.88 | 5.89 | -0.053 | YES | Loss of the α-helices as secondary structure |
| V1921L | - | 255957.86 | 5.89 | -0.055 | YES | - |
| G825R | TMD | 256042.97 | 5.91 | -0.056 | YES | Force the local backbone into an incorrect conformation and will disturb the local flexible torsion angles structure |
| A1773V | TMD | 255971.88 | 5.89 | -0.053 | YES | This size difference can affect the contacts with the lipid-membrane |
| H1838Y | TMD | 255969.86 | 5.87 | -0.054 | YES | Affect the hydrophobic interactions with the membrane lipids. loss of hydrogen bonds and/or disturbs correct folding |
| H1865Y | TMD | 255969.86 | 5.87 | -0.054 | NO | Loss of hydrogen bonds and/or disturbs correct folding |

**Abbreviation:** ECD = exocytoplasmic domain; NBD = nucleotide binding domain; TMD = transmembrane domain.

Compared to the wild type, the higher the value of the grand average of hydropathicity, the more hydrophobic the mutant is.

| **Appendix 2:** Supported evidences of four novel variants. | | | |
| --- | --- | --- | --- |
| **Variant** | **Criterion** | **Basis** | **Evidence** |
| c.1290dupC (p.W431fs) | PVS1 | Null variant (e.g., nonsense, frameshift, canonical ±1 or 2 splice sites, initiation codon, single exon or multiexon deletion) in a gene where LOF is a known mechanism of disease | Removal of the C-terminal conserved VFVNFA motif of ABCA4, which lead to a loss-of-function of ABCA transporter |
|  | PM2 | Absent from controls (or at extremely low frequency if recessive) in Exome Sequencing Project, 1000 Genomes Project, or Exome Aggregation Consortium | Absent from controls in 1000 Genomes Project, Exome Aggregation Consortium and Genome Aggregation Database |
|  | PM4 | Protein length changes as a result of in-frame deletion/insertions in a nonrepeat region or stop-loss variants | Mutation lead to code shift, which prematurely terminated protein translation |
|  | PP3 | Multiple lines of computational evidence support a deleterious effect on the gene or gene product (conservation, evolutionary, splicing impact, etc.) | It was considered “Deleterious” as predicted by function prediction software |
|  | PP4 | Patient’s phenotype or family history is highly specific for a diease with a single genetic etiology | The patient with typical genotype of CORD3 |
| c.2967dupT (G990fs) | PVS1 | Null variant (e.g., nonsense, frameshift, canonical ±1 or 2 splice sites, initiation codon, single exon or multiexon deletion) in a gene where LOF is a known mechanism of disease | Removal of the C-terminal conserved VFVNFA motif of ABCA4, which lead to a loss-of-function of ABCA transporter |
|  | PM2 | Absent from controls (or at extremely low frequency if recessive) in Exome Sequencing Project, 1000 Genomes Project, or Exome Aggregation Consortium | Absent from controls in 1000 Genomes Project, Exome Aggregation Consortium and Genome Aggregation Database |
|  | PM4 | Protein length changes as a result of in-frame deletion/insertions in a nonrepeat region or stop-loss variants | Mutation lead to code shift, which prematurely terminated protein translation |
|  | PP3 | Multiple lines of computational evidence support a deleterious effect on the gene or gene product (conservation, evolutionary, splicing impact, etc.) | It was considered “Deleterious” as predicted by function prediction software |
|  | PP4 | Patient’s phenotype or family history is highly specific for a diease with a single genetic etiology | The patient with typical phenotype of CORD3 |
| c.G5761T (p.V1921L) | PM2 | Absent from controls (or at extremely low frequency if recessive) in Exome Sequencing Project, 1000 Genomes Project, or Exome Aggregation Consortium | Absent from controls in 1000 Genomes Project, Exome Aggregation Consortium and Genome Aggregation Database |
|  | PM5 | Novel missense change at an amino acid residue where a different missense change determined to be pathogenic has been seen before | A mutation to “Methionine” was found at this position. The effect of this variant is annotated as: V > M (in dbSNP: rs61753032) [PMID:10958763] |
|  | PP3 | Multiple lines of computational evidence support a deleterious effect on the gene or gene product (conservation, evolutionary, splicing impact, etc.) | It was considered “Deleterious” as predicted by function prediction software |
|  | PP4 | Patient’s phenotype or family history is highly specific for a diease with a single genetic etiology | The patient with typical phenotype of STGD1 |
| c.C170G (p.P57R) | PM2 | Absent from controls (or at extremely low frequency if recessive) in Exome Sequencing Project, 1000 Genomes Project, or Exome Aggregation Consortium | Absent from controls in 1000 Genomes Project, Exome Aggregation Consortium and Genome Aggregation Database |
|  | PP3 | Multiple lines of computational evidence support a deleterious effect on the gene or gene product (conservation, evolutionary, splicing impact, etc.) | It was considered “Deleterious” as predicted by function prediction software |
|  | PP4 | Patient’s phenotype or family history is highly specific for a diease with a single genetic etiology | The patient with typical phenotype of CORD3 |
